# Reducing Energy Requirements in Future Bioregenerative Life Support Systems (BLSSs): Performance and Bioactive Composition of Diverse Lettuce Genotypes Grown Under Optimal and Suboptimal Light Conditions

**DOI:** 10.3389/fpls.2019.01305

**Published:** 2019-10-30

**Authors:** Youssef Rouphael, Spyridon A. Petropoulos, Christophe El-Nakhel, Antonio Pannico, Marios C. Kyriacou, Maria Giordano, Antonio Dario Troise, Paola Vitaglione, Stefania De Pascale

**Affiliations:** ^1^Department of Agricultural Sciences, University of Naples Federico II, Portici, Italy; ^2^Department of Agriculture, Crop Production and Rural Environment, University of Thessaly, Volos, Greece; ^3^Department of Vegetable Crops, Agricultural Research Institute, Nicosia, Cyprus

**Keywords:** bioactive compounds, bioregenerative food systems, carotenoids, *Lactuca sativa* L., light intensity, nitrate, polyphenols, space life support systems

## Abstract

Space farming for fresh food production is essential for sustaining long-duration space missions and supporting human life in space colonies. However, several obstacles need to be overcome including abnormal light conditions and energy limitations in maintaining Bioregenerative Life Support Systems (BLSSs). The aim of the present study was to evaluate six lettuce cultivars (baby Romaine, green Salanova, Lollo verde, Lollo rossa, red oak leaf and red Salanova) of different types and pigmentations under optimal and suboptimal light intensity and to identify the most promising candidates for BLSSs. Baby Romaine performed better than the rest of the tested cultivars under suboptimal light intensity, demonstrating a more efficient light-harvesting mechanism. Stomatal resistance increased under suboptimal light conditions, especially in the case of Lollo verde and red oak leaf cultivars, indicating stress conditions, whereas intrinsic water-use efficiency was the highest in baby Romaine and red oak leaf cultivars regardless of light regime. Nitrate content increased under suboptimal light intensity, especially in the cultivars green Salanova and Lollo verde, while P and Ca accumulation trends were also observed in baby Romaine and Lollo verde cultivars, respectively. Chicoric acid was the major detected phenolic acid in the hydroxycinnamic derivatives sub-class, followed by chlorogenic, caffeoyl-tartaric and caffeoyl-meso-tartaric acids. Chicoric and total hydroxycinnamic acids were not affected by light intensity, whereas the rest of the detected phenolic compounds showed a varied response to light intensity. Regarding cultivar response, red oak leaf exhibited the highest content in chicoric acid and total hydroxycinnamic acids content under suboptimal light intensity, whereas red Salanova exhibited the highest hydroxycinnamic derivatives profile under optimal light conditions. The main detected carotenoids were β-cryptoxanthin and violaxanthin+neoxanthin, followed by lutein and β-carotene. All the target carotenoids decreased significantly under low light intensity, while red Salanova maintained a distinct carotenoids profile. Overall, cultivation of assorted lettuce cultivars is the optimal scenario for space farming, where baby Romaine could provide adequate amounts of fresh biomass owing to its high light-use efficiency while red oak leaf and red Salanova could contribute to the daily dietary requirements for health-promoting bioactive compounds such as polyphenols and carotenoids.

## Introduction

The rapidly increasing population and the depletion of natural resources along with ongoing climate change have created uncertainty about food security or even human survival on Earth (Turchin and Green, 2018). Space colonization has been proposed as an alternative solution by pioneer aerospace scientists for decades (Chunxiao and Hong, 2008; Bamsey et al., 2009). For this purpose, several space programs have focused on life support systems in space through the construction of orbital colonies or the colonization of adjacent planets such as Mars (Zabel et al., 2016; Walker and Granjou, 2017). Although major breakthroughs have been achieved regarding space engineering and spaceflights during the last decades, the main issue that hinders space life is the use of higher plants in life support systems that could sustain human survival under unfavorable environments (Chunxiao and Hong, 2008; Graham and Bamsey, 2016; Dixon et al., 2017; Meinen et al., 2018).

So far, life in space flights and short missions have been supported by dried staple food or nutritional formulas, since fresh food production under space conditions remains a challenge due to several environmental constraints and a lack of knowledge of plant physiology under such conditions (Kyriacou et al., 2017; Meinen et al., 2018). Controlled Ecological Life Support Systems (CELSSs) or Bio-regenerative Life Support Systems (BLSSs) have been proposed as efficient means to ensure long-term human survival during space exploration through the sustainable provision of necessary food sources (Guo et al., 2017). The main idea behind these systems is based on Earth biosphere principles and aims to combine food crops and decomposers for the continuous supply of water and oxygen to space colonists without resupplying from Earth (Zabel et al., 2016). Current research programs such as MELiSSA (Micro-Ecological Life Support System Alternative) have focused on creating space habitats that can support human life through the autonomous supply of water, air and food (Walker and Granjou, 2017). Up to now, however, the most advanced CELSSs can only achieve regeneration of water and oxygen without being able to support food production under space conditions (Guo et al., 2017). Therefore, there is an urgent need to engineer a space biosphere where fresh food production from higher plants can sustain human life for long-term expeditions and space colonies. The major principles of plant cultivation in space environments are CO_2_ assimilation and O_2_ generation, as well as food production that can cover the daily nutrient requirements of colonizers and clean water production through plant transpiration.CO_2_ assimilation and O_2_ generation, as well as food production that can cover the daily nutrient requirements of colonizers and clean water production through plant transpiration. (Janik et al., 1989; Monje et al., 2003). Growing fresh food in space from vegetables species has been recognized as a key element for the success of CELSSs, because of their importance in human nutrition and their perishable nature (Kyriacou et al., 2017). Moreover, cultivation of higher plants in space missions has been associated with the better well-being of crew members, including mental and overall health status (Koga and Iwasaki, 2013; Guo et al., 2017).

BLSSs are supposed to support crew members’ needs in regard to food and nutrient requirements, however this has to be achieved under abnormal conditions, such as exposure to high levels of cosmic radiation, lack of a unilateral gravity vector, extreme temperatures, gas exchange related issues, growth adapted to limited chamber space, reduced nutrient sustainability and lack of convection. (Kuang et al., 2000; Monje et al., 2003; Kyriacou et al., 2017). A major challenge for adapting Earth-based agricultural practices aboard a spacecraft or in planetary bases is reduced gravity (or microgravity), which impacts fluid and gas distribution around the plants (Kuang et al., 2000). Moreover, low radiation levels (≤ 200-300 μmol m^-2^ s^-1^) are also among the serious constraints imposed on space farming as supplemental lighting is considered a highly energy-demanding subsystem of the space farm (Salisbury and Bugbee, 1988; Kyriacou et al., 2017). Therefore, Earth-based experiments within fully controlled chambers or artificial and closed ecological biospheres such as Biosphere 2 are valuable tools for testing plant and human responses to specific constraints, and the obtained results could be developed further to find application in future space missions (Haeuplik-Meusburger et al., 2014). Biosphere 2 is a mini-biospheric system of 1.27 ha and is isolated from its surroundings such as air and water, which makes it a prototype facility for perpetual life system need for space habitation (Nelson et al., 1992).

So far, various species (i.e., tuber crops, cereals, fruit and leafy vegetables) have been tested as potential candidates for food production in space. The selection criteria for these species were their adaptability under environmental constraints such as low light intensity, small plant size, high nutritional value and harvest index (Chunxiao and Hong, 2008; Wheeler, 2017). Moreover, space restrictions and energy input requirements (i.e. light) for plant production are of major importance in space food-production systems and are considered as selection criteria for candidate crops (Meinen et al., 2018).

Research on the identification of possible species that could support human life in space environments started in the early 1960s, and several experiments have been carried out so far (Chunxiao and Hong, 2008). According to the results of an initial survey of the acceptance of fresh vegetable crop candidates by space station crew members, lettuce (*Lactuca sativa* L.) was the most preferable crop among the various leafy greens tested (Mauerer et al., 2017). Its leaves constitute a nutritious food source, it is well-established in human diets, and when consumed in large quantities it could fulfill the recommended daily intake of most macro- and micro-nutrients (Mou, 2012). The nutritional value and bioactive compounds content of lettuce can be regulated within proper environmental conditions (e.g. light intensity and spectrum, nutrient solution composition, atmospheric CO_2_ conditions etc.), while the great availability of cultivars with very diverse qualities has proven to be the key to this species successful cultivation in space farms (Konstantopoulou et al., 2010; Park et al., 2012; Kang et al., 2014).

Light intensity is associated with several quality parameters of lettuce since it regulates the biosynthesis of secondary metabolites and affects the visual appearance of leaves (Zhou et al., 2009; Becker et al., 2013; Becker et al., 2014; Becker and Kläring, 2016; Pérez-López et al., 2018). According to Zhou et al. (2009), low light conditions (200-350 µmol m^-2^ s^-1^) resulted in low-quality lettuce leaves compared to high light (1000-1200 µmol m^-2^ s^-1^), which was attributed to the induction of antioxidant mechanisms when plants were subjected to higher than normal light intensities. Moreover, Kitazaki et al. (2018) suggested a metabolomics reprogramming approach for the effect of light intensity on the biosynthetic pathways of flavonoids and phenylpropanoids. By contrast, Urrestarazu et al. (2016) reported that even low light intensities (95 and 117 µmol m^-2^ s^-1^) can provide sufficient plant growth and high energy efficiency in lettuce, as it is considered a low-light adapted species (Zhen and van Iersel, 2017). The selection of cultivar is equally important since a significant variation in chemical composition and antioxidant compounds content has been reported among lettuce cultivars (López et al., 2013; Kim et al., 2018).

Considering the constraints that plants have to face when cultivated in space environment the aim of the present study was to evaluate the morpho-physiological performance as well as the chemical composition (mineral profile, lipophilic and hydrophilic antioxidant compounds) of six lettuce cultivars belonging to three different types (Romaine, butterhead and leaf lettuce) based on leaf shape and color, grown under two light conditions (optimal and low light intensity). The experiment was carried out in a Fitotron growth chamber in a closed soilless system using the nutrient film technique (NFT). The data obtained will assist scientists in discerning the genotypes that constitute the most suitable candidates for incorporation into BLSSs and space farming.

## Materials and Methods

### Standards and Chemicals

Acetonitrile, methanol water and dichloromethane (Merck; Darmstadt, Germany) were used for liquid chromatography diode array detection (LC-DAD) analysis and liquid chromatography tandem mass spectrometry (LC-MS/MS). Ethanol absolute and chloroform were obtained from VWR Chemicals (Radnor, PA). Hexane, butylated hydroxytoluene (BHT), formic acid (99% for mass spectrometry) along with analytical standards (chicoric acid, chlorogenic acid, lutein, β-carotene, violaxanthin, neoxanthin, β-cryptoxanthin, and cyanidin) were purchased from Sigma-Aldrich (St. Louis, MO). Ultrapure water was obtained from a Milli-Q Gradient A10 water purification system.

### Growth Chamber Environmental Control, Plant Material and Closed Soilless System Management

Two consecutive experiments were conducted in a Fitotron open-gas-exchange growth chamber (28 m^2^: 7.0 m × 2.1 m × 4.0 m; W × H × D; Process-C5, Spagnol srl, Treviso, Italy), at the experimental station of the Department of Agricultural Sciences, University of Naples Federico II, Italy.

For light treatments, High Pressure Sodium (HPS) lamps were used with two different light intensities, namely (i) optimal light intensity conditions at 420 µmol m^-2^ s^-1^ photosynthetic photon flux density (PPFD) (Experiment 1) and (ii) low light intensity conditions at 210 µmol m^-2^ s^-1^ PPFD (Experiment 2). Light intensity treatments (Experiments 1 and 2) were applied at a light/dark regime of 12/12 h, while temperature was regulated at 24/18°C for light and dark conditions respectively. Relative humidity (RH) was regulated at 65-75%. The experiment was carried out at ambient CO_2_ concentration (390 ± 20 ppm), while air exchange and dehumidification were guaranteed by two Heating, Ventilation and Air Conditioning (HVAC) systems. Hourly mean values of air temperature and RH recorded inside the Fitotron open-gas-exchange growth chamber during both experiments are provided as **Supplementary Material 1**.

Six lettuce (*Lactuca sativa* L.) cultivars belonging to three main lettuce types based upon leaf color and shape (Romaine [*Lactuca sativa* L. var. *longifolia*], butterhead [*Lactuca sativa* L. var. *capitata*] and leaf lettuce [*Lactuca sativa* L. var. *crispa*]), were used in both experiments. Common and scientific names, lettuce types and color as well as seed source are reported in supplementary information (**Supplementary Table S1**).

In both experiments, seedlings were transplanted at the two-true leaf stage in rockwool cubes (7 cm × 7 cm × 7 cm) (Delta, Grodan, Roermond, The Netherlands). Plant intra-row and inter-row spacing was 0.15 m and 0.43 m respectively, accounting for a total plant density of 15.5 plants m^-2^. Lettuce plants were cultivated in a Nutrient Film Technique (NFT) growing system (closed loop system). The NFT gullies were 200 cm long, 14.5 cm wide and 8 cm deep, having 1% inclination. Each gully was covered with propylene taps to avoid the evaporation. The flow rate of the nutrient solution was 1.5 L min^-1^, and it was supplied at the top end of each NFT channel and allowed to run slowly down the trough. The excess of the nutrient solution was collected in 25 L polypropylene tanks (one tank per experimental unit). The composition of the nutrient solution was: 8.0 mM N-NO_3_^-^, 1.5 mM S, 1.0 mM P, 3.0 mM K, 3.0 mM Ca, 1.0 mM Mg, 1.0 mM NH_4_^+^, 15 µM Fe, 9 µM Mn, 0.3 µM Cu, 1.6 µM Zn, 20 µM B, and 0.3 µM Mo. The electrical conductivity and pH of the nutrient solutions were monitored daily. The EC of the nutrient solution in the polypropylene tanks was kept within the range of 1.4 ± 0.1 dS m^-1^ by adding an additional volume of newly prepared nutrient solution when necessary. Moreover, the pH was maintained between 5.7 and 6.1 (5.9 ± 0.2) by adding an acid mixture with the same anionic ratio to the nutrient solution. In both growth chamber experiments (optimal and low light intensity experiments), a randomized-complete block design with three replicates was adopted to compare six lettuce cultivars (baby Romaine, green Salanova, Lollo rossa, Lollo verde, red oak leaf or red Salanova). Each experimental unit consisted of one NFT gully with twelve plants each (*n* = 216 lettuce plants for each experiment). The harvesting of all experimental units was performed 19 days after transplantation (DAT).

### Sampling, Morphological and Physiological Parameters

The first and last plant of each experimental unit in the NFT gullies were set as guards and were not included in the sampling for morphological, physiological and chemical composition parameters. Three plants per experimental unit were directly frozen in liquid nitrogen and stored at -80°C for further qualitative analysis, while seven plants per replicate were harvested in order to determine leaf number and measure fresh weight and leaf area, the latter being measured by an electronic area meter (LI-COR 3100C Biosciences, Lincoln, Nebraska, USA). Fresh lettuce samples from each experimental unit were dried in a forced-air oven at 70°C until constant weight (3 d) for dry weight per plant and dry matter content evaluation. Light Use Efficiency (LUE) was also calculated by dividing dry biomass with cumulative daily intercepted PPFD and expressed as g mol^-1^.

In both experiments, just before harvesting, the leaf gas exchange measurements were carried out on fully expanded leaves, using eight replicates for each of the lettuce cultivars. The net carbon dioxide assimilation rate (A_CO2_), stomatal resistance (r_s_) and transpiration rate (E) were recorded with a portable gas exchange analyzer (LCA-4; ADC BioScientific Ltd., UK) equipped with a broadleaf chamber (cuvette window area of 6.25 cm^2^) (Rouphael et al., 2017b). PPFD, RH and CO_2_ concentrations were set at ambient values (420 ± 10 and 210 ± 10 μmol m^−2^ s^−1^ for optimal and low light intensity conditions and RH 68 ± 2% and 390 ± 5 ppm respectively) and the flow rate of air was 400 ml s^−1^. Intrinsic Water Use Efficiency (WUE_i_) was calculated by dividing A_CO2_ by E (Carillo et al., 2019).

### Mineral Composition Analysis

Mineral and nitrate content was recorded in oven-dried lettuce leaf samples according to the method previously described by Rouphael et al. (2017a). In particular, dried leaf samples were ground with an electrical mill to a 841 µm mesh and 0.25 g of dry tissue was suspended in 50 ml of ultrapure water (Milli-Q, Merck Millipore, Darmstadt, Germany). Samples were then subjected to a freeze-thaw with liquid N and incubated at 80°C for 10 min in a shaking water bath (ShakeTemp SW22, Julabo, Seelbach, Germany). This process was repeated four times. The suspensions were centrifuged at 6000 rpm for 10 min (R-10 M, Remi Elektrotechnik Limited, India), and the supernatants were filtered with Whatman filter paper (0.20 μm; Whatman International Ltd., Maidstone, U.K.). Ion chromatography (ICS-3000, Dionex, Sunnyvale, CA, USA) coupled to a conductivity detector was implemented for NO_3_-N, P, K, Ca, Mg and Na analysis. The implemented columns were: a) IonPac CG12A (4 × 250 mm, Dionex, Corporation) guard column and IonPac CS12A (4 × 250 mm, Dionex, Corporation) analytical column for K, Ca, Mg and Na determination, b) IonPac AG11-HC (4 × 50 mm) guard column and IonPac AS11-HC (4 × 250 mm) analytical column for nitrate and P analysis. Next, 25 μL from filtered extracts were injected into the columns and eluted at a flow rate of 2 mL min^-1^ in isocratic mode for 15 min. The corresponding solvents were NaOH 5 mM for nitrate and P and CH_4_O_3_S 20 mM for K, Ca, Mg and Na. Standard curves for anions and cations in the range 0.05-0.5 mM were used for mineral content in tested samples and results were expressed as g kg^-1^ dry weight (dw), whereas nitrate was expressed as mg kg^-1^ on a fresh weight (fw) basis, according to the dry matter content (%) of each sample.

### Extraction and Quantification of Total Ascorbic Acid and Lipophilic Antioxidant Activity Analysis

For total ascorbic acid assessment the method described by Kampfenkel et al. (1995) was used. Total ascorbic acid content was determined on the basis of Fe^3+^ reduction to Fe^2+^ by ascorbic acid and the detection of Fe^2+^ complexes with 2,2-dipyridyl. Samples were pre-incubated in dithiothreitol to reduce dehydroascorbate to ascorbic acid and the latter was determined spectrophotometrically at 525 nm. For quantification of total ascorbic acid content, calibration curves of standard ascorbate were used, and the results were expressed as mg 100 g^-1^ fw.

Lipophilic antioxidant activity (LAA) was extracted from freeze-dried leaves (0.2 g) with methanol, and antioxidant activity of this extract was measured with the 2,20-azinobis 3-ethylbenzothiazoline-6-sulfonic acid ABTS method (Pellegrini et al., 1999). The principle of the assay is that the inhibitory response of the radical cation is proportional to the antioxidant concentration, and the reaction is complete at the time point of 2.5 min. The absorbance of the solutions was measured at 734 nm. LAA was expressed as mmol of Trolox (6-hydroxy-2,5,7,8-tetramethylchro man-2-carboxylic acid) per 100 g of dw (Fogliano et al., 1999).

### Hydroxycinnamic Acids and Anthocyanins Identification and Quantification

Hydroxycinnamic acids were extracted according to the method described by Llorach et al. (2008). Freeze-dried samples (400 mg) were extracted in a mixture of methanol/water/formic acid (50/45/5, v/v/v, 12 ml), followed by sonication for 30 min and centrifugation (2,500 × *g* for 30 min at 4°C). The supernatants were collected and centrifuged at 21100 × *g* for 15 min at 4°C and again collected and filtered through 0.22 µm cellulose filters (Phenomenex) before analysis. Hydroxycinnamic acid derivatives and anthocyanins were separated by a reversed phase C18 column (Prodigy, 250 × 4.6 mm, 5 µm, Phenomenex, Torrance, CA) equipped with a C18 security guard (4.0 × 3.0 mm, Phenomenex). The mobile phases were: (A) water formic acid (95:5, v/v) and (B) methanol through the following gradient of solvent B, (t in [min]/[%B]): (0/5), (25/40), (32/40). The flow rate was 1 mL min^-1^, and 20 µL of each extract was injected; the LC column was installed onto a binary system (LC-10AD, Shimadzu, Kyoto, Japan), equipped with a diode array detector (DAD, SPD-M10A, Shimadzu) and a Series 200 auto sampler (Perkin Elmer, Waltham, MA). Calibration curves at 330 nm of analytical standards of chlorogenic acid and di-caffeoyl-tartaric acid (chicoric acid) were used to quantify the corresponding compounds; the chicoric acid calibration curve was also used for caffeoyl-tartaric acid and caffeoyl-meso-tartaric acid quantification. Identification of all monitored hydroxycinnamic acids was performed by liquid chromatography tandem mass spectrometry (LC-MS/MS). The chromatographic profiles of reference curves and samples were recorded in multiple reaction monitoring mode (MRM) by using an API 3000 triple quadrupole (ABSciex, Carlsbad, CA). Negative electrospray ionization was used for detection, and source parameters were selected as follows: spray voltage: -4.2 kV; capillary temperature: 400°C; dwell time: 100 ms; and nebulizer gas and cad gas: 10 and 12, respectively (arbitrary units). Target compounds [M-H]^-^ were analyzed using mass transitions given in parentheses: chicoric acid (*m/z* 473→311, 293), chlorogenic acid (*m/z* 353→191), caffeoyl-tartaric acid (*m/z* 311→179, 149, retention time 15.8 min) and caffeoyl-meso-tartaric acid (*m/z*311→179, 149, retention time 17.8 min). The content of target polyphenols was expressed as mg 100 g^-1^ dw. Anthocyanins were identified by comparing the absorption spectra at 520 nm and the retention times of eluted peaks with those of cyanidin that was used as standard compound. Thus, total anthocyanins were reported, and results were expressed as μg cyanidin equivalent per g of samples on a dw basis.

### Target Carotenoids Extraction and Quantification

Carotenoids were assessed according to the method previously described by Vallverdú-Queralt et al. (2013) with slight modifications. A total of 100 mg of freeze-dried lettuce was added to a mixture of ethanol/hexane (4:3, v/v, 2.5 mL) with 1% Butylated hydroxytoluene (BHT), and the suspension was vortexed at 22°C for 30 s, sonicated for 5 min light protected, centrifuged (2500 × *g* at 4°C for 10 min) and filtered (0.45 µm nylon filters; Phenomenex, Torrance, CA). The supernatant was collected in a volumetric flask, and the same extraction procedure was repeated three times. The total amount of extracts of each sample was dried with a nitrogen air flow and stored at -20°C until further analysis. Dried extracts were re-dissolved in 1% BHT in chloroform, and 20 µL of each sample was injected into a C18 column (Prodigy, 250 × 4.6 mm, 5 µm, Phenomenex, Torrance, CA) equipped with a C18 security guard (4.0 × 3.0 mm, Phenomenex). Mobile phases were: (A) acetonitrile, hexane, methanol and dichloromethane (4:2:2:2, v/v/v/v) and (B) acetonitrile. The flow rate was 0.8 mL min^-1^ through the following gradient of solvent B (t in [min]/[%B]): (0/70), (20/60), (30/30) and (40/2). The same LC and auto sampler system described above was used for carotenoids quantitation, while for the identification and quantification of peaks analytical standards of violaxanthin, neoxanthin, β-cryptoxanthin, lutein and β-carotene were used for the comparison of absorbance spectra and retention times of eluted compounds at 450 nm. Intra- and inter-day assays were performed in triplicates and calibration curves were built accordingly. A recovery test was also performed by spiking two random samples with two known amounts of carotenoids (50 and 100 µg mL^-1^) and taking into account overestimation due to the presence of the target analytes in the samples. Concentrations of target carotenoids were reported as µg g^-1^ of samples on a dw basis.

### Statistical Analysis

The Shapiro–Wilk and Kolmororov–Smirnov procedures were performed to verify that the data had a normal distribution, and the Levene, O’Brien and Bartlet tests were conducted to verify the homogeneity of variances. All experimental data (**Supplementary Material 2**) were subjected to analysis of variance (ANOVA) using the SPSS 20 software package for Windows 2010. Combined analysis of variance was performed using light intensity conditions as a fixed variable (Gomez and Gomez, 1983). Means comparison was performed using the Duncan’s test at *P* ≤ 0.05.

## Results

### Implications of Light Intensity and Cultivars for Morphological and Physiological Parameters

The functional quality as well as the morpho-physiological parameters of *Lactuca sativa* L. were governed by genetic material and Genotype × Environment interaction. In this study, for most of the morphometric parameters measured, significant interactions were observed between the tested factors (Cultivar: C; Light intensity: L) (**Table 1**). Leaf area, leaf number and fresh and dry biomass were higher under optimal light conditions with a varied response in terms of the cultivars. In particular, red Salanova presented the highest leaf area, while leaf number was the highest for both green and red Salanova cultivars (**Table 1**). Regarding fresh yield, the lowest values were reported for green Salanova, Lollo rossa and red oak leaf cultivars under low light conditions (**Table 1**). Interestingly, fresh yield reduction varied between the tested cultivars; all cultivars, however, performed worse under low light compared to optimal light conditions. In fact, decreasing light intensity from 420 to 210 µmol m^-2^ s^-1^ PPFD decreased the fresh yield of baby Romaine, green Salanova, Lollo rossa, Lollo verde, red oak leaf and red Salanova by 36.8%, 64.6%, 65.4%, 54.6%, 58.5% and 60.3%, respectively (**Table 1**). When shoot dry mass was expressed in g plant^-1^, the recorded values were higher for Lollo verde and red Salanova cultivars under optimal light conditions, whereas when dry matter was expressed as a percentage for red oak leaf and baby Romaine, it exhibited the highest values at both optimal and low light intensity conditions, respectively (**Table 1**).

**Table 1:** Growth parameters, fresh biomass, dry biomass and leaf dry matter percentage of lettuce grown hydroponically in a Fitotron open-gas-exchange growth chamber in relation to light conditions and cultivar.

Source of variance	Leaf area (cm^2^ plant^-1^)	Leaf number (no. plant^-1^)	Fresh biomass (g plant^-1^)	Dry biomass (g plant^-1^)	Dry matter (%)
Cultivar (C)					
Baby Romaine	799 ± 84 d	23.76 ± 1.72 c	61.0 ± 6.2 b	3.41 ± 0.38 a	5.58 ± 0.07 a
Green Salanova	1037 ± 143 b	45.55 ± 5.01 a	60.5 ± 13.0 b	2.83 ± 0.55 c	4.82 ± 0.13 c
Lollo rossa	715 ± 138 e	13.30 ± 0.62 d	61.7 ± 13.5 b	2.91 ± 0.62 c	4.78 ± 0.05 c
Lollo verde	893 ± 133 c	13.81 ± 0.48 d	64.2 ± 11.1 ab	3.33 ± 0.59 a	5.20 ± 0.11 b
Red oak leaf	1035 ± 151 b	37.21 ± 2.88 b	51.9 ± 9.8 c	2.82 ± 0.55 c	5.41 ± 0.09 ab
Red Salanova	1245 ± 214 a	44.28 ± 4.70 a	66.0 ± 12.8 a	3.09 ± 0.56 b	4.76 ± 0.09 c
Light intensity (μmol m^−2^ s^−1^) (L)					
420 (Optimal)	1271 ± 63 a	35.21 ± 4.23 a	85.4 ± 2.3 a	4.26 ± 0.07 a	5.06 ± 0.11
210 (Low)	637 ± 29 b	24.09 ± 2.31 b	36.4 ± 1.5 b	1.87 ± 0.09 b	5.12 ± 0.07
C × L					
Baby Romaine × Optimal	983 ± 23 d	27.40 ± 0.95 e	74.7 ± 1.2 b	4.24 ± 0.10 b	5.69 ± 0.05 a
Green Salanova × Optimal	1355 ± 19 b	56.67 ± 1.31 a	89.5 ± 0.9 a	4.06 ± 0.06 b	4.58 ± 0.10 f
Lollo rossa × Optimal	1019 ± 48 d	14.53 ± 0.52 g	91.7 ± 3.7 a	4.28 ± 0.14 b	4.68 ± 0.04 ef
Lollo verde × Optimal	1177 ± 81 c	14.80 ± 0.31 g	88.3 ± 5.0 a	4.63 ± 0.19 a	5.29 ± 0.07 bc
Red oak leaf × Optimal	1368 ± 29 b	43.20 ± 2.23 b	73.4 ± 3.5 b	4.03 ± 0.13 b	5.52 ± 0.10 ab
Red Salanova x Optimal	1722 ± 33 a	54.67 ± 1.38 a	94.6 ± 2.3 a	4.33 ± 0.15 ab	4.57 ± 0.05 f
Baby Romaine × Low	614 ± 32 f	20.12 ± 0.81 f	47.2 ± 2.0 c	2.57 ± 0.08 c	5.46 ± 0.08 ab
Green Salanova × Low	718 ± 11 ef	34.42 ± 0.22 c	31.6 ± 0.3 e	1.59 ± 0.04 e	5.06 ± 0.14 cd
Lollo rossa × Low	411 ± 15 g	12.07 ± 0.34 g	31.7 ± 1.3 e	1.54 ± 0.05 e	4.88 ± 0.04 def
Lollo verde × Low	610 ± 30 f	12.82 ± 0.27 g	40.1 ± 2.2 d	2.04 ± 0.06 d	5.10 ± 0.21 cd
Red oak leaf × Low	701 ± 35 ef	31.22 ± 0.84 d	30.4 ± 1.9 e	1.61 ± 0.12 e	5.30 ± 0.14 bc
Red Salanova × Low	768 ± 16 e	33.90 ± 0.79 cd	37.5 ± 0.7 d	1.85 ± 0.03 de	4.94 ± 0.04 de
Significance					
C	***	***	***	***	***
L	***	***	***	***	ns
C × L	***	***	***	***	**

NS, **, *** Non-significant or significant at P ≤ 0.01, and 0.001, respectively. Different letters within each column indicate significant differences at P ≤ 0.05. All data are expressed as mean ± SE, n = 3.

**Figure 1:**
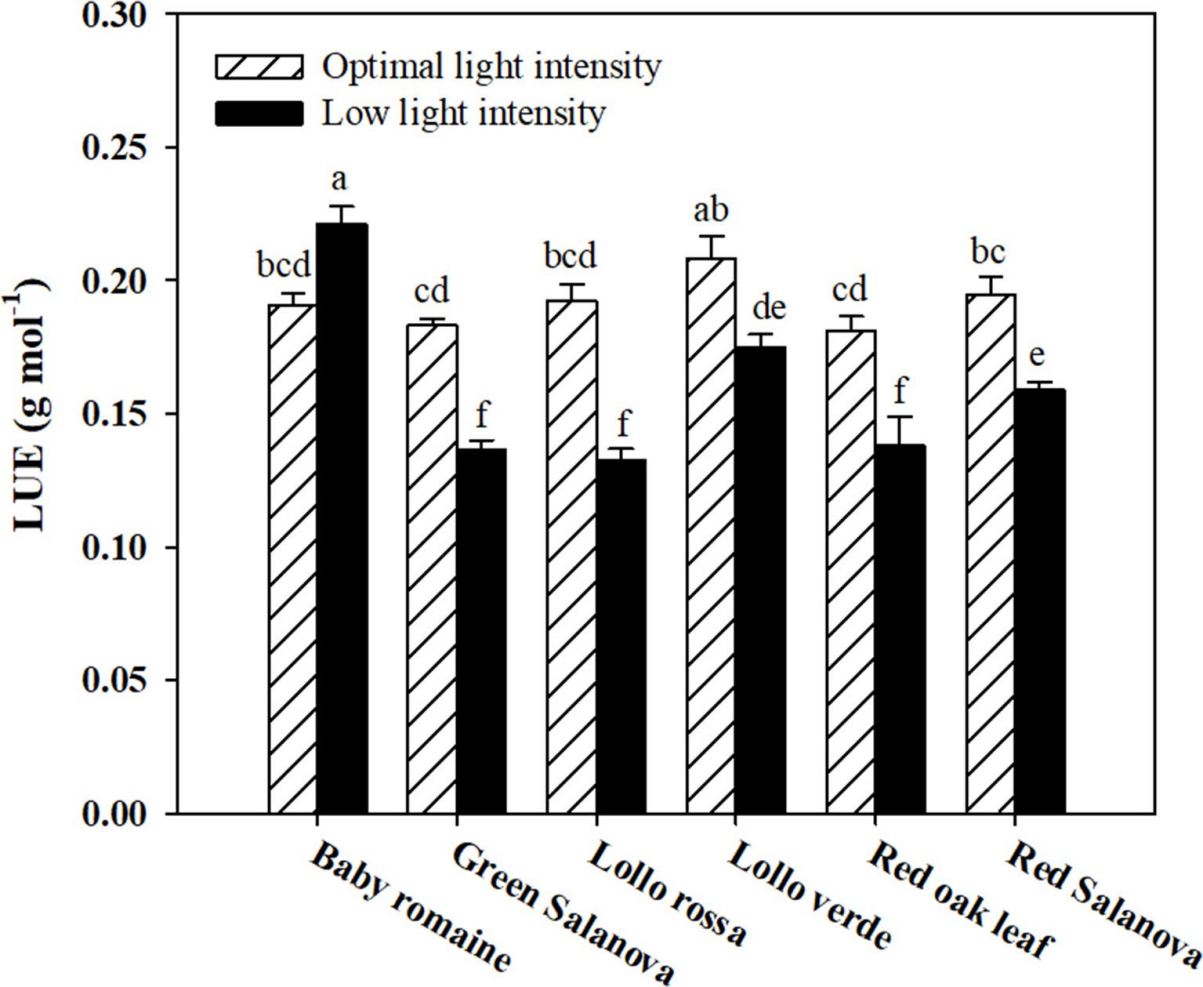
Light Use Efficiency (LUE) of lettuce grown hydroponically in a Fitotron open-gas-exchange growth chamber in relation to light conditions and cultivar. All data are expressed as mean ± SE, *n* = 3. Different letters above each bar indicate significant differences at *P* ≤ 0.05.

Significant interactions between the two tested factors were also observed in the studied physiological parameters, except for the transpiration rate (E), which was affected by C and L factors without significant interaction between them (**Table 2**). The net CO_2_ assimilation rate (A_CO2_) was the highest under optimal light conditions with red Salanova exhibiting the highest overall values. A contrasting trend was observed for stomatal resistance (r_s_), which increased under low light intensity conditions, especially in the case of Lollo verde and red oak leaf cultivars (**Table 2**). On the other hand, when averaged over cultivars, E values were higher by 51.3% under optimal light conditions, while, in regards to cultivar effect, the highest E rate was observed for red Salanova plants without being significantly different from baby Romaine and green Salanova cultivars (**Table 2**). Regarding intrinsic water use efficiency (WUE_i_), the highest values were recorded for baby Romaine and red oak leaf cultivars, regardless of light regime, followed by red Salanova under optimal light. Finally, the highest light use efficiency (LUE) was detected in baby Romaine plants when grown under low light intensity conditions, without, however, being significantly different from Lollo verde plants grown under optimal light conditions (**Figure 1**).

**Table 2:** Physiological parameters [net CO_2_ assimilation rate (A_CO2_); stomatal resistance (r_s_); transpiration rate (E); intrinsic Water Use Efficiency (WUE_i_)] of lettuce grown hydroponically in a Fitotron open-gas-exchange growth chamber in relation to light conditions and cultivar.

Source of variance	A_CO2_ (μmol CO_2_ m^−2^ s^−1^)	r_s_ (m^2^ s^1^ mol^−1^)	E (mol H_2_O m^−2^ s^−1^)	WUEi (μmol CO_2_ mol^−1^ H_2_O)
Cultivar (C)				
Baby Romaine	10.49 ± 0.45 a	5.00 ± 0.41 cd	2.60 ± 0.13 ab	4.08 ± 0.11 b
Green Salanova	7.85 ± 0.49 c	5.15 ± 0.51 cd	2.67 ± 0.13 ab	2.93 ± 0.12 d
Lollo rossa	5.99 ± 0.56 d	5.92 ± 0.62 b	2.51 ± 0.15 b	2.36 ± 0.16 e
Lollo verde	4.06 ± 0.43 e	5.72 ± 0.93 bc	2.26 ± 0.16 c	1.75 ± 0.13 f
Red oak leaf	9.55 ± 0.59 b	8.03 ± 0.57 a	2.21 ± 0.16 c	4.40 ± 0.15 a
Red Salanova	10.07 ± 0.80 a	4.94 ± 0.65 d	2.76 ± 0.16 a	3.58 ± 0.11 c
Light intensity (μmol m^−2^ s^−1^) (L)				
420 (Optimal)	9.99 ± 0.37 a	3.70 ± 0.21 b	2.98 ± 0.06 a	3.39 ± 0.13 a
210 (Low)	5.76 ± 0.32 b	8.15 ± 0.26 a	1.97 ± 0.05 b	2.96 ± 0.17 b
C × L				
Baby Romaine × Optimal	12.06 ± 0.30 b	3.64 ± 0.23 de	3.03 ± 0.12	4.03 ± 0.17 ab
Green Salanova × Optimal	9.57 ± 0.32 c	3.36 ± 0.29 def	3.05 ± 0.15	3.19 ± 0.17 c
Lollo rossa × Optimal	8.01 ± 0.24 e	4.08 ± 0.30 d	2.93 ± 0.14	2.79 ± 0.16 cd
Lollo verde × Optimal	5.59 ± 0.15 g	2.33 ± 0.15 f	2.79 ± 0.13	2.03 ± 0.11 e
Red oak leaf × Optimal	11.68 ± 0.27 b	6.24 ± 0.44 c	2.73 ± 0.15	4.38 ± 0.24 ab
Red Salanova x Optimal	13.01 ± 0.31 a	2.53 ± 0.14 ef	3.35 ± 0.05	3.90 ± 0.12 b
Baby Romaine × Low	8.72 ± 0.08 d	6.53 ± 0.35 c	2.12 ± 0.08	4.14 ± 0.14 ab
Green Salanova × Low	5.91 ± 0.12 g	7.16 ± 0.25 bc	2.25 ± 0.05	2.64 ± 0.10 d
Lollo rossa × Low	3.71 ± 0.23 h	7.99 ± 0.76 b	2.05 ± 0.14	1.89 ± 0.19 ef
Lollo verde × Low	2.33 ± 0.27 i	9.53 ± 0.51 a	1.66 ± 0.07	1.43 ± 0.19 f
Red oak leaf × Low	7.16 ± 0.21 f	10.05 ± 0.50 a	1.63 ± 0.07	4.42 ± 0.18 a
Red Salanova × Low	6.76 ± 0.21 f	7.65 ± 0.22 b	2.09 ± 0.01	3.23 ± 0.11 c
Significance				
C	***	***	***	***
L	***	***	***	***
C × L	***	***	ns	**

NS, **, *** Non-significant or significant at P ≤ 0.05, 0.01, and 0.001, respectively. Different letters within each column indicate significant differences at P ≤ 0.05. All data are expressed as mean ± SE, n = 3.

### Implications of Light Intensity and Cultivars for Nitrate and Mineral Profile

Nitrate content and mineral composition as a function of cultivar and light intensity are presented in **Table 3**. Low light intensity resulted in an increase of nitrate content, especially in the case of green Salanova and Lollo verde cultivars. Among the minerals analyzed, K was by far the most abundant, regardless of the cultivar and light intensity treatment, ranging from 58.2 to 70.9 g kg^-1^ dw, followed by Ca (4.8-13.5 g kg^-1^ dw), P (4.7-7.7 g kg^-1^ dw), Mg (2.0-3.0 g kg^-1^ dw) and finally Na (0.3-0.8 g kg^-1^ dw) (**Table 3**).

**Table 3:** Nitrate, phosphorus (P), potassium (K), calcium (Ca), magnesium (Mg) and sodium (Na) concentrations of lettuce grown hydroponically in a Fitotron open-gas-exchange growth chamber in relation to light conditions and cultivar.

Source of variance	Nitrate (mg kg^-1^ fw)	P (g kg^-1^ dw)	K (g kg^-1^ dw)	Ca (g kg^-1^ dw)	Mg (g kg^-1^ dw)	Na (g kg^-1^ dw)
Cultivar (C)						
Baby Romaine	2494 ± 54 b	6.28 ± 0.62 a	64.26 ± 1.30	6.73 ± 0.88 cd	2.56 ± 0.09 bc	0.33 ± 0.04
Green Salanova	2699 ± 136 ab	5.86 ± 0.22 b	68.00 ± 1.36	9.71 ± 0.77 b	2.31 ± 0.19 d	0.37 ± 0.03
Lollo rossa	2081 ± 160 c	5.56 ± 0.26 bc	65.03 ± 3.22	6.14 ± 0.49 d	2.62 ± 0.09 bc	0.36 ± 0.06
Lollo verde	2806 ± 177 a	5.16 ± 0.19 cd	62.04 ± 1.18	10.94 ± 1.16 a	2.91 ± 0.08 a	0.55 ± 0.14
Red oak leaf	2084 ± 34 c	5.08 ± 0.17 d	60.44 ± 1.10	7.44 ± 0.69 c	2.70 ± 0.06 b	0.31 ± 0.01
Red Salanova	2108 ± 114 c	5.61 ± 0.17 bc	66.43 ± 2.65	6.01 ± 0.43 d	2.45 ± 0.11 cd	0.30 ± 0.02
Light intensity (μmol m^−2^ s^−1^) (L)						
420 (Optimal)	2275 ± 56 b	5.16 ± 0.10 b	65.15 ± 1.08	6.27 ± 0.37 b	2.66 ± 0.05 a	0.41 ± 0.05
210 (Low)	2482 ± 124 a	6.02 ± 0.21 a	63.58 ± 1.35	9.38 ± 0.58 a	2.56 ± 0.08 b	0.33 ± 0.01
C × L						
Baby Romaine × Optimal	2419 ± 90 cde	4.90 ± 0.03 de	66.56 ± 1.76	4.78 ± 0.14 h	2.38 ± 0.07 cd	0.32 ± 0.08
Green Salanova × Optimal	2423 ± 38 cde	5.42 ± 0.13 cd	65.08 ± 0.60	8.05 ± 0.32 cd	2.65 ± 0.08 abcd	0.33 ± 0.02
Lollo rossa × Optimal	1974 ± 69 e	5.68 ± 0.11 bc	66.19 ± 1.65	5.14 ± 0.16 gh	2.67 ± 0.04 abc	0.42 ± 0.13
Lollo verde × Optimal	2461 ± 61 cd	4.82 ± 0.16 de	60.55 ± 2.05	8.42 ± 0.53 c	2.96 ± 0.09 a	0.76 ± 0.23
Red oak leaf × Optimal	2152 ± 4.0 cde	4.71 ± 0.05 e	62.71 ± 0.82	5.96 ± 0.40 fg	2.70 ± 0.11 abc	0.30 ± 0.02
Red Salanova x Optimal	2223 ± 221 cde	5.42 ± 0.32 cd	69.81 ± 4.77	5.30 ± 0.59 gh	2.61 ± 0.11 abcd	0.31 ± 0.03
Baby Romaine × Low	2570 ± 29 bc	7.65 ± 0.18 a	61.95 ± 0.17	8.68 ± 0.20 c	2.74 ± 0.07 abc	0.33 ± 0.06
Green Salanova × Low	2975 ± 121 ab	6.31 ± 0.17 b	70.92 ± 0.65	11.38 ± 0.34 b	1.98 ± 0.02 e	0.40 ± 0.04
Lollo rossa × Low	2189 ± 335 cde	5.44 ± 0.55 cd	63.87 ± 6.91	7.13 ± 0.42 de	2.57 ± 0.20 bcd	0.30 ± 0.02
Lollo verde × Low	3151 ± 185 a	5.50 ± 0.20 cd	63.52 ± 0.77	13.47 ± 0.17 a	2.86 ± 0.13 ab	0.35 ± 0.02
Red oak leaf × Low	2015 ± 31 de	5.45 ± 0.08 cd	58.18 ± 0.51	8.93 ± 0.11 c	2.71 ± 0.05 abc	0.32 ± 0.02
Red Salanova × Low	1993 ± 60 e	5.80 ± 0.10 bc	63.04 ± 0.90	6.72 ± 0.31 ef	2.30 ± 0.15 de	0.30 ± 0.01
Significance						
C	***	***	ns	***	***	ns
L	**	***	ns	***	*	ns
C × L	**	***	ns	***	**	ns

NS,*,**, *** Non-significant or significant at P ≤ 0.05, 0.01, and 0.001, respectively. Different letters within each column indicate significant differences at P ≤ 0.05. All data are expressed as mean ± SE, n = 3.

Neither the cultivar nor the light intensity regime had a significant effect on K and Na concentration in lettuce leaves (avg. 64.4 and 0.4 g kg^-1^ dw, respectively). Regarding mineral content, a cultivar-dependent response to light conditions was observed, with P and Ca content being the highest for baby Romaine and Lollo verde plants grown under low light intensity conditions respectively (**Table 3**). By contrast, Mg content increased under optimal light conditions without significant differences for most of the tested cultivars between the applied light regimes. The sole exceptions to this were green and red Salanova plants (**Table 3**).

### Implications of Light Intensity and Cultivars for Hydrophilic and Lipophilic Antioxidant Compounds and Antioxidant Capacity

Concentrations of hydroxycinnamic acids and anthocyanins are presented in **Table 4**. It shows that total concentration of hydroxycinnamic acids differed between lettuce cultivars, light intensity regimes and their combinations. Chicoric acid (di-caffeoyl-tartaric acid) was the most abundant compound, followed by chlorogenic acid, whereas caffeoyl-tartaric and caffeoyl-meso-tartaric acids were present at lower amounts (**Table 4**). Significant differences in individual and hydroxycinnamic acid content were observed mostly concerning the cultivar effect, whereas light conditions had an impact only on the less abundant compounds and not on chicoric acid and total hydroxycinnamic acids content. Red oak leaf was the most affected cultivar by low light intensity, showing the highest content for most of the individual phenolic acids, including chicoric and chlorogenic acids, and consequently for their total content (**Table 4**). Interestingly, red Salanova, which was the second-best cultivar in regard to chicoric and total hydroxycinnamic acids content, was beneficially affected by optimal light conditions (highest concentrations of caffeoyl tartaric, chlorogenic, chicoric and caffeoyl-meso-tartaric acids) and thus indicated a cultivar-dependent response of lettuce to light regime (**Table 4**). Expectedly, anthocyanins were only detected in red-pigmented lettuce cultivars (Lollo rossa, red oak leaf and red Salanova) (**Table 4**). High light intensity in particular increased anthocyanins by 273.9% in red Salanova, whereas an opposite trend was observed for Lollo rossa and red oak leaf with a significant decrease of anthocyanins by 59.6% and 46.7% respectively (**Table 4**).

**Table 4:** Hydroxycinnamic acids composition, total hydroxycinnamic acids and anthocyanins of lettuce grown hydroponically in a Fitotron open-gas-exchange growth chamber in relation to light conditions and cultivar.

Source of variance	Caffeoyl tartaric acid (mg 100 g^-1^ dw)	Chlorogenic acid (mg 100 g^-1^ dw)	Chicoric acid (mg 100 g^-1^ dw)	Caffeoyl-meso-tartaric acid (mg 100 g^-1^ dw)	∑ hydroxycinnamic acids (mg 100g^-1^ dw)	Anthocyanins (μg cyanidin eq. g^-1^ dw)
Cultivar (C)						
Baby Romaine	6.04 ± 0.52 bc	4.09 ± 0.79 c	26.7 ± 3.95 b	1.22 ± 0.40 d	38.0 ± 5.51 c	n.d.
Green Salanova	3.97 ± 0.67 c	2.91 ± 0.06 c	16.1 ± 2.94 b	0.43 ± 0.07 d	23.4 ± 3.58 c	n.d.
Lollo rossa	9.43 ± 1.75 a	14.58 ± 1.86 c	86.0 ± 5.98 a	6.12 ± 1.81 c	116.2 ± 8.23 b	6.22 ± 1.45 b
Lollo verde	7.40 ± 1.28 ab	3.00 ± 0.68 c	31.4 ± 3.14 b	0.58 ± 0.09 d	42.3 ± 5.01 c	n.d.
Red oak leaf	8.70 ± 2.69 a	66.19 ± 20.94 a	106.9 ± 30.36 a	11.45 ± 3.45 b	193.3 ± 57.19 a	16.35 ± 2.23 a
Red Salanova	4.79 ± 0.46 c	48.03 ± 6.03 b	96.8 ± 21.58 a	21.73 ± 6.40 a	171.3 ± 33.86 a	6.25 ± 1.67 b
Light intensity (μmol m^−2^ s^−1^) (L)						
420 (Optimal)	4.58 ± 0.41 b	18.42 ± 5.12 b	56.1 ± 11.60	8.61 ± 3.09 a	87.7 ± 19.54	n.d
210 (Low)	8.86 ± 1.01 a	27.84 ± 9.55 a	65.2 ± 12.83	5.23 ± 1.62 b	107.1 ± 24.17	n.d
C × L						
Baby Romaine × Optimal	5.30 ± 0.69 de	2.92 ± 0.49 e	20.1 ± 1.92 cd	0.67 ± 0.25 e	29.0 ± 2.75 fg	n.d.
Green Salanova × Optimal	2.58 ± 0.28 e	3.01 ± 0.08 e	9.8 ± 0.56 d	0.30 ± 0.05 e	15.6 ± 0.82 g	n.d.
Lollo rossa × Optimal	6.29 ± 1.06 cde	18.13 ± 1.96 cde	89.7 ± 7.77 b	10.10 ± 0.71 c	124.2 ± 9.80 c	3.58 ± 0.78 c
Lollo verde × Optimal	5.24 ± 0.64 de	1.62 ± 0.37 e	26.6 ± 1.66 cd	0.44 ± 0.14 e	33.9 ± 2.15 fg	n.d.
Red oak leaf × Optimal	3.05 ± 0.41 de	24.53 ± 5.77 cd	45.9 ± 8.01 c	5.33 ± 1.59 cde	78.8 ± 15.58 cdef	11.46 ± 0.58 b
Red Salanova × Optimal	4.99 ± 1.00 de	60.34 ± 4.85 b	144.5 ± 7.08 a	34.83 ± 5.75 a	244.6 ± 18.26 b	9.87 ± 0.95 b
Baby Romaine × Low	6.78 ± 0.57 cd	5.26 ± 1.24 de	33.2 ± 5.64 cd	1.76 ± 0.67 e	47.0 ± 7.97 efg	n.d.
Green Salanova × Low	5.35 ± 0.49 de	2.81 ± 0.01 e	22.4 ± 1.74 cd	0.56 ± 0.06 e	31.1 ± 1.91 fg	n.d.
Lollo rossa × Low	12.57 ± 2.07 ab	11.03 ± 0.96 de	82.4 ± 10.23 b	2.13 ± 0.16 de	108.1 ± 13.31 cd	8.87 ± 1.70 b
Lollo verde × Low	9.56 ± 1.78 bc	4.39 ± 0.52 de	36.1 ± 4.92 cd	0.72 ± 0.05 e	50.7 ± 7.08 defg	n.d.
Red oak leaf × Low	14.35 ± 2.05 a	107.85 ± 20.56 a	168.0 ± 28.59 a	17.56 ± 4.43 b	307.8 ± 54.83 a	21.24 ± 0.80 a
Red Salanova × Low	4.58 ± 0.19 de	35.72 ± 2.63 c	49.1 ± 2.38 c	8.62 ± 0.07 cd	98.0 ± 5.10 cde	2.64 ± 0.21 c
Significance						
C	***	***	***	***	***	**
L	***	*	ns	*	ns	*
C × L	***	***	***	***	***	***

NS,*,**, *** Non-significant or significant at P ≤ 0.05, 0.01, and 0.001, respectively. n.d., not detected. Different letters within each column indicate significant differences at P ≤ 0.05. All data are expressed as mean ± SE, n = 3.

**Figure 2:**
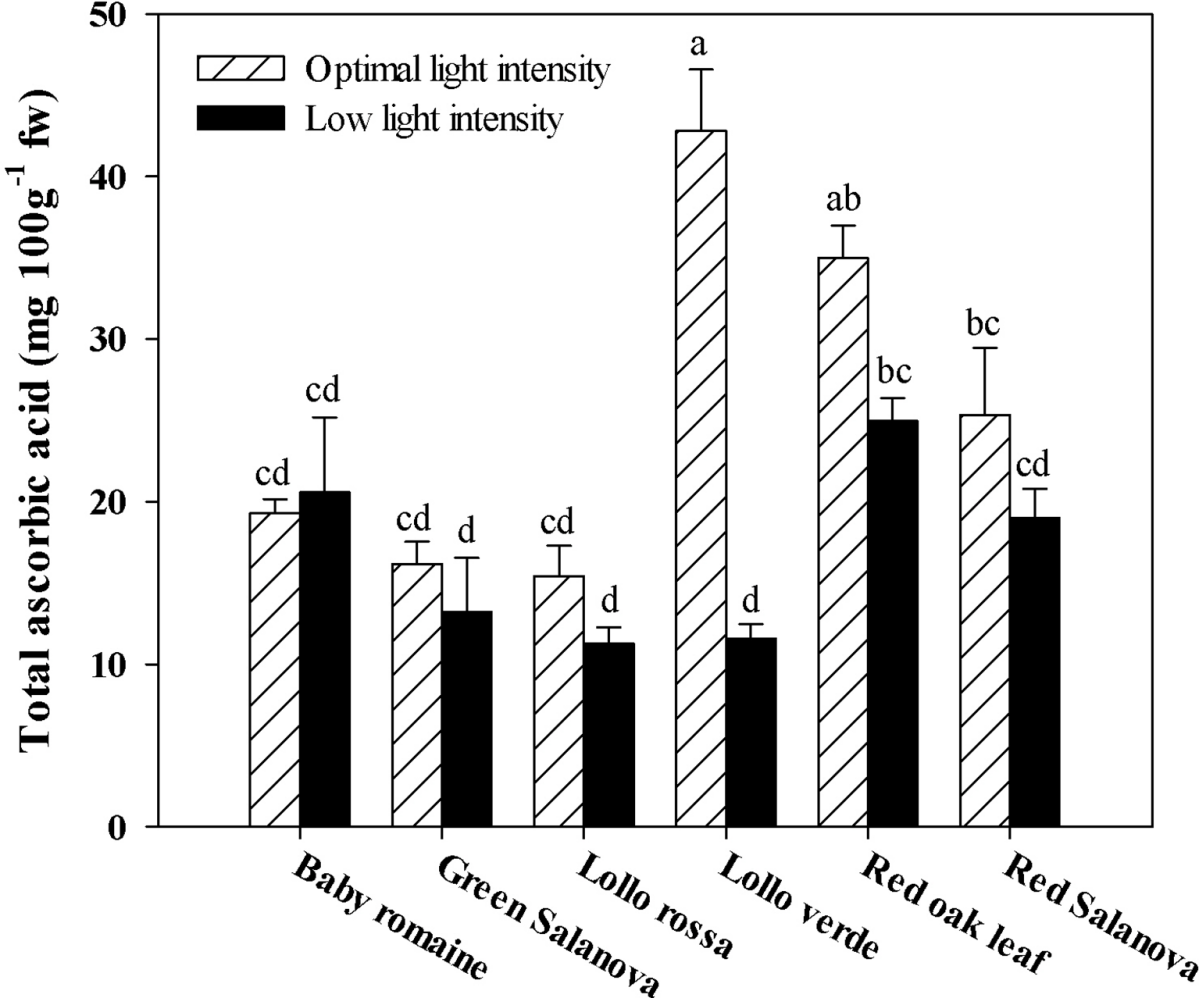
Total ascorbic acid concentration in lettuce grown hydroponically in a Fitotron open-gas-exchange growth chamber in relation to light conditions and cultivar. All data are expressed as mean ± se, *n* = 3. Different letters above each bar indicate significant differences at *P* ≤ 0.05.

Similarly to hydroxycinnamic acids, total ascorbic acid was significantly affected by the two tested factors (data not shown). In most cases, ascorbic acid content was highly affected by light conditions. Particularly, in Lollo verde the reduction of light intensity from 420 to 210 µmol m^-2^ s^-1^ PPFD had a detrimental effect on ascorbic acid content, resulting in a four-fold reduction compared to optimal conditions (**Figure 2**).

**Figure 3:**
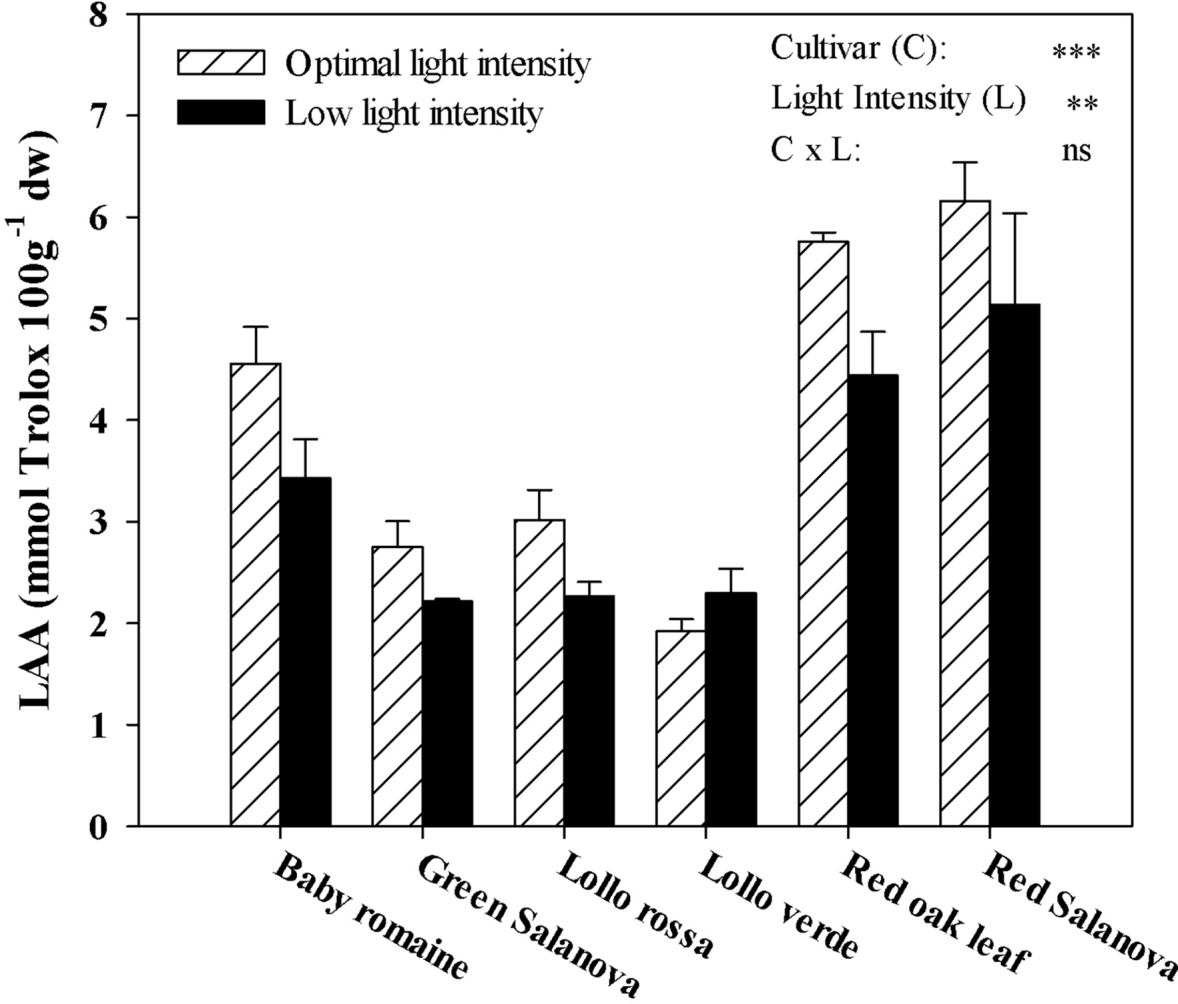
Effects of light conditions and cultivar on lipophilic antioxidant activity (LAA) of lettuce grown hydroponically in a Fitotron open-gas-exchange growth chamber in relation to light conditions and cultivar. All data are expressed as mean ± SE, n = 3. Ns, **, *** Non-significant or significant at P ≤ 0.01, and 0.001, respectively.

LAA was significantly affected by cultivars and light intensity, but not by their interaction (**Figure 3**). As for the light intensity treatment mean effect averaged over cultivars, LAA was beneficially affected by optimal light conditions (+22.1%) compared to a low light regime (**Figure 3**). Regardless of the light intensity treatment, red Salanova and red oak leaf cultivars had the highest LAA content, followed by baby Romaine (**Figure 3**).

In the current study, all the carotenoids detected are presented in **Table 5**. The main pigments detected were β-cryptoxanthin and violaxanthin + neoxanthin, followed by lutein and β-carotene (**Table 5**). Significant C × L interaction was noted with respect to the concentrations of carotenoids. Optimal light conditions had a beneficial effect on the biosynthesis of most of the detected soluble pigments in baby Romaine (violaxanthin + neoxanthin, lutein, β-cryptoxanthin and β-carotene) and red Salanova (violaxanthin + neoxanthin and lutein). Interestingly, violaxanthin + neoxanthin, lutein, β-cryptoxanthin and β-carotene concentrations were higher in red Salanova leaves grown under low light intensity compared to the remaining five cultivars (**Table 5**).

**Table 5:** Composition of carotenoids profile of lettuce grown hydroponically in a Fitotron open-gas-exchange growth chamber in relation to light conditions and cultivar.

Source of variance	Violaxanthin + neoxanthin (μg violaxanthin eq. g^-1^ dw)	Lutein (μg g^-1^ dw)	β-cryptoxanthin (μg g^-1^ dw)	β-carotene (μg g^-1^ dw)
Cultivar (C)				
Baby Romaine	843.7 ± 119 b	608.9 ± 61.6 b	1031.7 ± 95.5 ab	372.5 ± 79.2 b
Green Salanova	597.2 ± 53.3 c	242.8 ± 23.1 c	444.5 ± 19.5 c	163.1 ± 27.7 c
Lollo rossa	490.4 ± 31.1 d	237.8 ± 14.3 c	408.9 ± 26.4 c	191.4 ± 34.4 c
Lollo verde	431.4 ± 15.1 d	180.9 ± 7.10 d	257.3 ± 18.2 d	129.6 ± 16.6 d
Red oak leaf	831.9 ± 52.1 b	611.0 ± 38.6 b	947.1 ± 42.3 b	359.0 ± 28.9 b
Red Salanova	996.0 ± 19.1 a	739.2 ± 13.9 a	1046.2 ± 29.2 a	404.1 ± 24.4 a
Light intensity (μmol m^−2^ s^−1^) (L)				
420 (Optimal)	788.2 ± 58.2 a	485.7 ± 59.2 a	741.4 ± 89.9 a	346.0 ± 33.6 a
210 (Low)	608.6 ± 51.9 b	387.8 ± 50.8 b	637.2 ± 73.2 b	193.8 ± 24.6 b
C × L				
Baby Romaine × Optimal	1102.6 ± 55.3 a	746.0 ± 9.26 a	1228.9 ± 82.1 a	547.6 ± 27.5 a
Green Salanova × Optimal	707.3 ± 32.9 e	287.9 ± 18.0 e	479.0 ± 22.5 e	223.1 ± 14.1 e
Lollo rossa × Optimal	559.2 ± 7.10 fg	268.2 ± 9.90 ef	466.5 ± 13.1 e	267.3 ± 10.4 d
Lollo verde × Optimal	458.5 ± 12.0 gh	184.9 ± 11.6 g	233.8 ± 23.5 f	165.7 ± 8.98 f
Red oak leaf × Optimal	886.9 ± 61.6 cd	673.0 ± 43.5 b	986.6 ± 43.5 bc	417.5 ± 26.1 b
Red Salanova x Optimal	1014.8 ± 31.5 ab	754.5 ± 26.8 a	1053.4 ± 63.6 b	455.1 ± 16.6 b
Baby Romaine × Low	584.7 ± 27.0 f	471.8 ± 9.76 d	834.5 ± 4.88 d	197.4 ± 4.54 ef
Green Salanova × Low	487.1 ± 31.5 fgh	197.7 ± 18.0 g	410.0 ± 14.8 e	103.1 ± 6.38 g
Lollo rossa × Low	421.6 ± 9.08 h	207.3 ± 0.92 fg	351.3 ± 3.99 ef	115.4 ± 7.20 g
Lollo verde × Low	404.3 ± 16.2 h	176.9 ± 10.0 g	280.8 ± 23.4 f	93.5 ± 2.62 g
Red oak leaf × Low	776.8 ± 82.3 de	549.0 ± 41.7 c	907.5 ± 74.0 cd	300.5 ± 9.12 d
Red Salanova × Low	977.1 ± 21.9 bc	723.8 ± 3.75 ab	1039.0 ± 13.6 b	353.0 ± 9.91 c
Significance				
C	***	***	***	***
L	***	***	***	***
C × L	***	***	***	***

*** Significant at P ≤ 0.001, respectively. Different letters within each column indicate significant differences at P ≤ 0.05. All data are expressed as mean ± SE, n = 3.

## Discussion

Space colonization can only be achieved through the integration of controlled ecological life support systems (CELSSs) or bio-regenerative life support systems (BLSSs) in space biospheres that could support human life for long time periods without the replenishment of supplies from the Earth. However, space farming is a very challenging task as higher plants have to be grown under abnormal conditions and key obstacles have to be overcome. These obstacles mostly include environmental constraints (e.g. microgravity, low pressure, excessive radiation and so forth), space and energy limitations, as well as the need to ensure the optimal nutrition of crew members through the production of functional and bio-fortified foods (Guo et al., 2017; Kyriacou et al., 2017). Several crops have been suggested for space farming based on specific criteria, and lettuce has been identified as a candidate leafy vegetable crop in several research studies (Massa et al., 2017; Meinen et al., 2018). The present study evaluated the response of six lettuce cultivars under optimal and low light intensity in order to identify the most promising genotypes towards yield components, physiological parameters, mineral profile and bioactive compounds content.

Low light intensity had a detrimental effect on fresh biomass yield, regardless of the cultivar. However, baby Romaine plants performed better (a reduction of 36% and 39% on fresh and dry weight basis, respectively) than the rest of the tested cultivars under such conditions in terms of fresh and dry biomass yield (reduction between 55 to 65% on both fresh and dry basis), demonstrating that baby Romaine cv. showed physiological acclimation in response to their limited light environment (**Table 1**). This response could be explained by the higher A_CO2_ and WUE_i_, and lower r_s_ values recorded for baby Romaine cultivars under low light intensity conditions, indicating a more efficient light-harvesting mechanism (higher LUE) of the genotype under this specific environmental constraint (**Table 2** and **Figure 1**). These results are consistent with the study of Fu et al. (2012), who detected slight differences in the yield of romaine lettuce under similar light conditions, suggesting that LUE was the highest at 200 μmol m^-2^ s^-1^, as it was for baby Romaine plants in our study. Other researchers highlighted the effect of photoperiod, light quality and light intensity on lettuce growth and development, which altogether define light conditions (Kang et al., 2014; Muneer et al., 2014). Therefore, the light effect on biomass yield has multiple aspects to be considered, especially in space farming where every light parameter (photoperiod, quality and intensity) has to be optimized for higher biomass production and better LUE.

Light intensity affected nitrate and mineral content in a genotype-dependent manner (**Table 3**). As expected, low light intensity resulted in an increase in nitrate content, especially for green Salanova and Lollo verde cultivars, as nitrate reductase activity is associated with light intensity, among other factors (Petropoulos et al., 2011; Colla et al., 2018). Another putative mechanism behind the accumulation of nitrate under low light intensity could be that the key enzymes, such as glutamate synthase and glutamine synthetase, are inhibited, whereas asparagine synthetase involved in stabilizing nitrate for transport and storage is stimulated (Colla et al., 2018). However, even in the case of these two cultivars (green Salanova and Lollo verde) the detected nitrate content was within the limits set by the EU commission for safe lettuce consumption (EU regulation: No 1258/2011).

Under low photosynthetically active radiation conditions vegetables tend to concentrate key macronutrients, particularly P and Ca, in their storage organs as a result of lower crop productivity. In the current study, high fresh yield triggered by favorable environmental conditions (optimal light intensity) may have accelerated lettuce biomass accumulation, thus decreasing the macro-mineral (P and Ca) concentration due to a dilution effect (Pérez-López et al., 2015; Stagnari et al., 2015). Sufficient mineral intake through diet is fundamental for human health, and vegetable cultivation in space farming should aim to provide products with an enhanced mineral content. For this purpose, cation antagonism has to be considered attentively when adjusting the nutrient solution composition in order to combine the best agronomic performance with increased mineral and low nitrate content.

Chicoric acid (the most abundant of hydroxycinnamic acids) and total hydroxycinnamic acids were not affected by light intensity, whereas the rest of the detected hydroxycinnamic acids showed variable responses in relation to light intensity and cultivar (**Table 4**). This is an important finding of the study, since phenolic compounds are associated with health benefits and an increased intake from low amounts of food is a desirable feature when selecting species suitable for space farming. Only two of the detected hydroxycinnamic acids (caffeoyl tartaric acid and chlorogenic acid) increased under low light intensity, with a stronger light effect observed on the former, without, however, affecting the total content of hydroxycinnamic acids (**Table 4**). Both compounds were subject to cultivar × light interaction since significant increase under low light conditions was observed for caffeoyl tartaric acid only in three cultivars (Lollo rossa, Lollo verde, Red oak leaf) and for chlorogenic acid only in one cultivar (red Salanova). The increase observed in these two phenolic compounds, which are powerful antioxidants, may be an important mechanism upon which plants can rely to counterbalance reduction in antioxidant enzymatic activity and metabolites under low light intensity (Campa et al., 2017). As previously demonstrated, in plants grown under 210 µmol m^-2^ s^-1^ of light intensity, chloroplast redox status significantly decreased. This affected the accumulation of carotenoids, in particular violaxanthin + neoxanthin, lutein and β-carotene, as well as antioxidant enzymes activity (Jahns and Holzwarth, 2012). In addition to the pivotal role in light harvesting and in the dissipation of excess light energy, carotenoids function as Reactive Oxygen Species (ROS) scavengers and singlet oxygen molecule quenchers to minimize oxidative damage of photosynthetic apparatus and membrane lipids (Jahns and Holzwarth, 2012). Moreover, they act as ROS-mediated stress signals (Havaux, 2014). Their decrease at 210 µmol m^-2^ s^-1^ is, therefore, potentially deleterious as this can impair the plant antioxidant defense mechanisms, causing an increase in ROS and subsequent photo-damage, even under low light conditions that seemingly constitute non-significant stress. With the exception of caffeoyl tartaric acid and chlorogenic acid (which increased under low radiation intensity), similar results have been reported in the literature, where a reduction of radiation intensity (from a PPFD of 410 to 225 µmol m^-2^ s^-1^) did not affect either total phenolic acid or individual phenolic acid (chicoric and caffeoylmalic acids) content in red oak leaf lettuce (Becker et al., 2013). An explanation for the different responses in terms of chlorogenic acid could be the length of the growth cycle (3 versus 9 weeks). Moreover, light is an important factor affecting the synthesis and accumulation of anthocyanins, an important subgroup of flavonoids responsible for red color in lettuce (Maier and Hoecker, 2015; Kim et al., 2016). Several experiments have demonstrated that higher light intensity could promote the up-regulation of anthocyanin synthesis related genes, thus boosting anthocyanin accumulation (Zoratti et al., 2014; Guan et al., 2016; Zhang et al., 2016). In the current experiment, this was the case only for red Salanova cultivar, whereas light intensity at 420 µmol m^-2^ s^-1^ had a detrimental effect on anthocyanins in both Lollo rossa and red oak leaf cultivars (**Table 4**), suggesting the existence of some other light-dependent mechanism modulating anthocyanin synthesis and accumulation (Maier and Hoecker, 2015). In fact, the latter authors reported that the CONSTITUTIVE PHOTOMORPHOGENIC1 (COP1)/SUPPRESSOR OF PHYA (SPA) complex may not be fully inactivated under low light intensity, thereby increasing anthocyanin accumulation in lettuce leaves.

Genotype response also has to be considered, since the results showed a multi-response profile of the tested cultivars under different light regimes (**Table 4**). Similarly, the literature also suggests a great variation in phenolic compounds content among the various lettuce genotypes (Llorach et al., 2008; Bunning et al., 2010). Our findings suggested that all red-leaf lettuce cultivars tested had a distinct profile in hydroxycinnamic acids; under optimal light conditions, red Salanova in particular can be used as a nutrient-dense food. On the other hand, red oak leaf had the highest content of hydroxycinnamic derivatives when grown under low light intensity as a consequence of severe stress, as indicated also by the low fresh biomass yield and the high stomatal resistance (**Tables 1** and **2**, respectively). Therefore, although a high content of phenolic compounds might seem attractive, space farming constraints must also be considered, and the optimal ratio between quality and fresh biomass yield must be achieved.

The interest in fat-soluble pigments such as carotenoids is not recent, owing to their beneficial effects on human well-being, in particular human vision during future space missions (Kyriacou et al., 2017). Soluble pigments content, especially β-carotene (a precursor of vitamin A), zeaxanthin and lutein, which contribute to the vision protection of crew members from excessive radiation in space conditions, were higher when optimal light intensity was applied regardless of cultivar (**Table 5**). Similar results have been reported by Lefsrud et al. (2008) and Li et al. (2009), who showed that key carotenoids (β-carotene and lutein) in kale and spinach leaves were significantly higher under optimal (300 µmol m^-2^ s^-1^) than under low (100 µmol m^-2^ s^-1^) irradiation conditions. Furthermore, red Salanova contained consistently high amounts of pigments regardless of light intensity, which is an extremely important finding from a nutritional point of view due to the antioxidant properties of these compounds (Cohu et al., 2014; Kitazaki et al., 2018). Similar trends were observed for total ascorbic acid content and lipophilic antioxidants, which also decreased under low light intensity conditions (**Figures 2** and **3**, respectively). For almost all the tested genotypes no significant differences were observed between low and optimal light intensity, whereas only Lollo verde showed almost a fourfold decrease in total ascorbic acid content under low light intensity (**Figure 2**). Despite the fact that lettuce is not considered a rich source of ascorbic acid, the great variation among the existing cultivars allows for its complementary role to ascorbic acid intake along with other food sources (Bunning et al., 2010; Mampholo et al., 2016). So far, the literature has confirmed the effect of light quality on pigments content (Kołton et al., 2014; Ouzounis et al., 2015), as well the increase of anthocyanins under excessive light intensity as a protection mechanism for leaf chlorophylls (Feild et al., 2001). However, Fu et al. (2017) suggested that very low light intensity (60-140 μmol m^-2^ s^-1^) may also increase carotenoids content compared to higher light intensity (220 μmol m^-2^ s^-1^), a finding which has to be investigated further since energy saving through implementation of low light intensity is of major importance to space farming. Another approach was the one proposed by Cohu et al. (2014), who tested the effect of low light intensity supplemented with short intervals of high light intensity pulses and found that these short pulses may trigger carotenoids biosynthesis and zeaxanthin in particular in *Arabidopsis thaliana* plants. Such a practice could be very useful in space conditions where energy consumption is a key element. Moreover, Mampholo et al. (2016) reported a great variation in β-carotene among lettuce cultivars, which, according to Mou (2012), could be attributed partly to head structure and the function of β-carotene as a complement to chlorophyll’s light-harvesting compound. Therefore, the effect of light conditions in space environments has to be investigated further through the evaluation of various lettuce genotypes in order to find cultivars that are acclimatized to such conditions and where the final fresh produce has increased content in soluble pigments.

## Conclusion

Space farming for fresh food production is the next breakthrough to be achieved for the successful outcome of long-duration space missions and space colonization. However, cultivation of higher plants under space conditions entails the consideration of several parameters with contrasting effects on plant growth and physiology as well as produce quality. For example, space limitations and low light intensity in space shuttles or space stations requires high light-use efficiency without compromising fresh biomass yield and the quality of the final product. The results of our study supported the existing research reports that suggest lettuce as a candidate crop for space farming. The great variation among the existing cultivars allows for the selection of genotypes with highly efficient light-harvesting mechanisms in order to provide sufficient fresh biomass yield, while at the same time quality may increase through the increase of antioxidant compounds (such as soluble pigments, ascorbic acid and hydroxycinnamic derivative compounds) and the decrease of nitrate. Among the cultivars tested under low light intensity conditions, baby Romaine showed the best agronomic performance in terms of fresh biomass yield and physiological parameters. Moreover, the same cultivar contained an increased content of P and Ca under low light intensity, without nitrate content being affected by the light regime. Regarding the bioactive properties, red-colored cultivars such as red oak leaf and red Salanova had the highest content in phenolic derivatives and soluble pigments under low and optimal light intensity, respectively, while both of them showed the highest lipophilic antioxidant activity, regardless of light regime. In conclusion, the content of bioactive compounds in lettuce cultivars appears to be influenced strongly by the genetic material and light intensity. Therefore, specific cultivars and light condition combinations could be applied in separate growth chambers to obtain both the desired profile of functional compounds and the adequate amounts of fresh produce necessary to support human life in prolonged space missions or space stations.

## Data Availability Statement

The raw data supporting the conclusions of this manuscript will be made available by the authors, without undue reservation, to any qualified researcher.

## Author Contributions

YR: defined the scientific hypothesis, set up the experimental protocol, coordinated the research and he was involved in writing; SP: was significantly involved in writing the paper and discussing the experimental data; CE-N: run accurately both experiments, sampling and analysis, AP and MG: worked on mineral and statistical analysis as well as tables and figures preparation, AT and PV: performed the whole HPLC analysis and gave a contribution on the related *Materials and Methods* section; MK and SP: contributed in improving the manuscript.

## Conflict of Interest

The authors declare that the research was conducted in the absence of any commercial or financial relationships that could be construed as a potential conflict of interest.

## Supplementary Material

Supplementary Table 1 Common and scientific name, lettuce type, leaf color and seed source of the six lettuce (Lactuca sativa L.) cultivars considered in this study.

Supplementary Material 1 Hourly mean values of air temperature and relative humidity recorded inside the Fitotron open-gas-exchange growth chamber during both experiments.

Supplementary Material 2 All experimental data subjected to the analysis of variance (ANOVA).
