# Supplementary material for: Reducing Energy Requirements in Future Bioregenerative Life Support Systems (BLSSs): Performance and Bioactive Composition of Diverse Lettuce Genotypes Grown Under Optimal and Suboptimal Light Conditions
Source: Front Plant Sci. 2019 Oct 30;10:1305. doi: 10.3389/fpls.2019.01305 (PMC6831738; doi:10.3389/fpls.2019.01305)
Supplement: Supplementary Table 1 — Common and scientific name, lettuce type, leaf color and seed source of the six lettuce (Lactuca sativa L.) cultivars considered in this study. [file Table_1.doc]

**Table S1. Common and scientific name, lettuce type, leaf color and seed source of the six lettuce (*Lactuca sativa* L.) cultivars considered in this study.**

| Cultivar name | Scientific name | Lettuce type | Leaf color | Source |
| --- | --- | --- | --- | --- |
| Baby Romaine | *Lactuca sativa* L. var. *longifolia* | Romaine | Dark Green | Rijk Zwaan |
| Green Salanova | *Lactuca sativa* L. var. *capitata* | Butterhead | Light Green | Rijk Zwaan |
| Lollo rossa | *Lactuca sativa* L. var. *crispa* | Leaf lettuce | Red | Rijk Zwaan |
| Lollo verde | *Lactuca sativa* L. var. *crispa* | Leaf lettuce | Light green | Nunhems |
| Red oak leaf | *Lactuca sativa* L. var. *crispa* | Leaf lettuce | Red | Rijk Zwaan |
| Red Salanova | *Lactuca sativa* L. var. *capitata* | Butterhead | Red | Rijk Zwaan |
